# Publisher Correction: Network-based prediction of drug combinations

**DOI:** 10.1038/s41467-019-09692-y

**Published:** 2019-04-15

**Authors:** Feixiong Cheng, István A. Kovács, Albert-László Barabási

**Affiliations:** 1 Center for Complex Networks Research and Department of Physics, Northeastern University, Boston, MA 02115 USA; 2 Center for Cancer Systems Biology and Department of Cancer Biology, Dana-Farber Cancer Institute, Boston, MA 02215 USA; 3 Genomic Medicine Institute, Lerner Research Institute, Cleveland Clinic, Cleveland, OH 44106 USA; 4 Department of Molecular Medicine, Cleveland Clinic Lerner College of Medicine, Case Western Reserve University, Cleveland, OH 44195 USA; 5 Case Comprehensive Cancer Center, Case Western Reserve University School of Medicine, Cleveland, OH 44106 USA; 6 Channing Division of Network Medicine, Department of Medicine, Brigham and Women’s Hospital, Harvard Medical School, Boston, MA 02215 USA; 7 Center for Network Science, Central European University, Budapest, 1051 Hungary

Correction to: *Nature Communications* 10.1038/s41467-019-09186-x, published online 13 March 2019

The original version of this Article contained an error in Acknowledgements, which incorrectly omitted the following: ‘This work was also supported by NHLBI grant P01HL132825 to A.-L.B.’ This has been corrected in both the PDF and HTML versions of the Article.

