# Supplementary material for: Network-based prediction of drug combinations
Source: Nat Commun. 2019 Mar 13;10:1197. doi: 10.1038/s41467-019-09186-x (PMC6416394; doi:10.1038/s41467-019-09186-x)
Supplement: Supplementary file 1 — Supplementary Information [file 41467_2019_9186_MOESM1_ESM.pdf]

SUPPLEMENTARY INFORMATION FOR

Network-based Prediction of Drug Combinations

Cheng et al., *Nature Communications* 2019

*Corresponding author. Email: alb@neu.edu (A.L.B.)

Supplementary Information includes:

Supplementary Notes **1** to **5**.
Supplementary Figures **1** to **13**.
Supplementary Table **1.**
Supplementary Data **1** to **5**.
Supplementary References

Table of contents

Supplementary Notes

Supplementary Note 1.. 3
Supplementary Note 2.. 3
Supplementary Note 3.. 5
Supplementary Note 4.. 6
Supplementary Note 5.. 9

Supplementary Figures

Supplementary Fig. 1 11
Supplementary Fig. 2 12
Supplementary Fig. 3 13
Supplementary Fig. 4 14
Supplementary Fig. 5 15
Supplementary Fig. 6 17
Supplementary Fig. 7 18
Supplementary Fig. 8 19
Supplementary Fig. 9 19
Supplementary Fig. 10 20
Supplementary Fig. 11 21
Supplementary Fig. 12 22
Supplementary Fig. 13 23

Supplementary Table 1 24

Supplementary References 25

Supplementary Note 1

Network proximity of drug-disease pairs. Given the set of drug targets, *X*, and the set of disease proteins, *Y*, we first calculate the shortest path length *d(x,y)* between nodes *x* and *y* in the human protein-protein interactome, leading to the "closest" distance measure:

$$d(X,Y) = \frac{1}{\|Y\|}\sum_{y\in Y} min_{x\in X} d(x,y). \qquad (1)$$

The significance of this measure is evaluated by comparing to the reference distance distribution corresponding to the expected network topological distance between two randomly selected groups of proteins matched to size and degree (connectivity) distribution as the original disease proteins and drug targets in the human interactome. This procedure was repeated 1,000 times. The mean distance ($\overline{d}$) and standard deviation (σ_d) of the reference distribution were used to calculate a z-score (*z*) by converting an observed closest distance to a normalized distance as below.

$$z = \frac{d-\overline{d}}{\sigma_d} \qquad (2)$$

We calculate the z-score between drugs and diseases by randomly sampling both sets of nodes (drug targets and disease proteins) at the same time. The detailed description for z-score calculation was provided in the previous study[1]. The toolbox package for z-score calculation is available at github.com/emreg00/toolbox.

Supplementary Note 2

To build a comprehensive human protein-protein interactome, we assembled data from a total of 15 bioinformatics and systems biology databases with multiple experimental evidences. Specifically, we focused on high-quality protein-protein interactions (PPIs) with five types of experimental evidences: (i) Binary PPIs tested by high-throughput yeast-two-hybrid (Y2H) systems: we combined binary PPIs tested from two public available high-quality Y2H datasets[2,3] and one unpublished dataset, publicly available at: http://ccsb.dana-farber.org/ interactome-data.html; (ii) Kinase-substrate interactions by literature-derived low-throughput and high-throughput experiments from KinomeNetworkX[4], Human Protein Resource Database (HPRD)[5], PhosphoNetworks[6,7], PhosphositePlus[8], dbPTM 3.0[9], and Phospho. ELM[10]; (iii) Literature-curated PPIs identified by affinity purification followed by mass spectrometry (AP-MS), Y2H and by literature-derived low-throughput experiments from BioGRID[11], PINA[12], HPRD[5], MINT[13], IntAct[14], and InnateDB[15]; (iv) High-quality PPIs from protein three-dimensional (3D) structures reported in Instruct[16]; (v) Signaling network by literature-derived low-throughput experiments as annotated in SignaLink2.0[17]. The genes were mapped to their Entrez ID based on the NCBI database[18] as well as their official gene symbols based on GeneCards (http://www.genecards.org/). Duplicated pairs were removed. In this study, all inferred data, including evolutionary analysis, gene expression data, and metabolic associations, were excluded. The resulting updated human

interactome used in this study includes 243,603 PPIs connecting 16,677 unique proteins (**Supplementary Data 1**), offering over 40% increase in size compared to our previously used human interactome[19].

Supplementary Note 3

Collecting gold-standard pairwise drug combinations. *Drug Combination DataBase (DCDB).* DCDB is a manually curated database containing drug combination information from approximately 14,000 clinical studies[20]. DCDB (v2.0) contains 1,363 clinically reported drug combinations for 904 distinctive components[20].

Therapeutic Target Database (TTD). TTD is a comprehensive database providing the drug, target, and drug-target pathway information for facilitating drug discovery[21]. Current TTD (v5.1.01) includes 72 pharmacodynamically synergistic, 14 additive, and 4 antagonist combinations[21].

FDA Electronic Orange Book. FDA electronic orange book (http://www.accessdata.fda.gov/scripts/cder/ob/default.cfm) contains FDA-approved drug products, including drug combination information based on the basis of safety and effectiveness by the Federal Food, Drug, and Cosmetic Act.

Literatures. We manually collected experimentally validated or clinically reported drug combination information from the literatures and publicly available sources

(Drugs.com and eMedExpert [http://www.emedexpert.com/]) for several complex diseases, including hypertension[22-24], type-2 diabetes[25-27], and cancer[28-32].

In this study, we focus on pairwise drug combinations by assembling the clinical data from the abovementioned data sources. In addition, each drug in combinations was required to have the experimentally validated target information: each EC_{50}, IC_{50}, K_i, or $K_d \leq 10$ μM. Compound name, generic name or commercial name of each drug was standardized by MeSH and UMLS vocabularies[33] and further transferred to DrugBank ID from the DrugBank database (v4.3)[34]. Duplicated drug pairs were removed. In total, 681 unique pairwise drug combinations connecting 362 drugs were retained (**Supplementary Data 3**).

Supplementary Note 4

Collecting disease-association genes. We integrated disease-gene annotation data from 8 bioinformatics data sources as described below.

OMIM: The OMIM database (Online Mendelian Inheritance in Man: http://www.omim.org/)[35] is a comprehensive collection covering literature-curated human disease genes with various high-quality experimental evidences.

CTD: The Comparative Toxicogenomics Database (http://ctdbase.org/)[36] provides information about interactions between chemicals and gene products, and their association with various diseases. Here, only manually curated gene-disease

interactions from the literatures were used.

ClinVar: ClinVar is a public archive of relationships among sequence variation and various human phenotypes (https://www.ncbi.nlm.nih.gov/clinvar/)[37]. Here, only clinically significant relationships among variants and cardiovascular phenotypes annotated in ClinVar were used.

GWAS Catalog: The NHGRI-EBI Catalog of published genome-wide association studies (GWAS: https://www.ebi.ac.uk/gwas/)[38] provides unbiased SNP-disease associations with genome-wide significance. Here, data were downloaded from the website: https://www.ebi.ac.uk/gwas/ (access in December 2015) and a SNP on a specific disease with a genome-wide significance ($p < 5.0 \times 10^{-8}$) was used.

GWASdb: GWASdb includes a more comprehensive data curation and knowledge integration for SNP-trait associations from GWAS for PubMed and other resources[39]. Here, the curated moderate SNP-disease associations ($p < 1.0 \times 10^{-3}$) annotated in GWASdb were used.

PheWAS Catalog: PheWAS Catalog contains SNP-trait associations identified by the phenome-wide association study (PheWAS) paradigm within electronic medical records, an unbiased approach to replication and discovery that interrogates relationships between targeted genotypes and multiple phenotypes[40]. Here, the SNP-disease associations at phenome-wide significance ($p < 0.05$) were downloaded from the PheWAS Catalog website: phewas.mc.vanderbilt.edu.

HuGE Navigator: HuGE Navigator is an integrated disease candidate gene

database based on the core data from PubMed abstracts using text mining algorithms[41]. Here, the literature-reported disease-gene annotation data with known PubMed IDs from HuGE Navigator were used.

DisGeNET: DisGeNET is a comprehensive database for collecting disease-associated genes[42]. In 2015, DisGeNET contained over 380,000 associations connecting over 16,000 genes and 13,000 diseases by integrating expert-curated databases and text-mining data. Here, only expert-curated data were used.

We assembled disease-gene annotation data from 8 different resources and excluded the duplicated entries. We annotated all protein-coding genes using gene Entrez ID, chromosomal location, and the official gene symbols from the NCBI database[18]. Each cardiovascular event was defined by Medical Subject Headings (MeSH) and Unified Medical Language System (UMLS) vocabularies (https://www.nlm.nih.gov/mesh/MBrowser.html)[33]. In this study, we constructed disease-associated genes for 4 types of cardiovascular events: arrhythmia (MeSH ID: D001145), heart failure (MeSH ID: D006333), myocardial infarction (MeSH ID: D009203), and hypertension/high blood pressure (MeSH ID: D006973). The detailed description of disease-gene annotation data integration from different data sources is provided in our previous study[19].

Supplementary Note 5: Permutation tests

A permutation test, also called a randomization test, is a standard test to compute statistical significance[43]. The null hypothesis of the permutation test is generated by counting all possible values of the test statistic under rearrangements of the true labels (e.g., experimentally validated drug combinations or clinically reported adverse drug-drug interactions) on the shuffled data points (all possible drug pairs). Specifically, the statistical significance is given by:

$$P = \frac{\#\{S_m(p) < S_m\}}{\#\{total\ permutations\}} \quad (3)$$

A nominal *P* is computed for each network configuration (P1-P6) by counting the number of shuffled differences (S_m) more than true difference ($S_m(p)$). For the “drug combinations” column in **Fig. 2**, we collected in total 65 hypertensive drug combinations from three types of experimental evidence: (i) FDA-approved evidence, (ii) data from the Clinicaltrials.gov database, and (iii) Preclinical studies from the literature (**Supplementary Data 3**). When quantifying the network-based relationship between two drug-target modules and a disease module (drug-drug-disease combinations), we find that there is only one hypertensive drug combination in the Overlapping Exposure category (**Fig. 2a**) while 59 combinations fall into the Complementary Exposure category (**Fig. 2b**). We randomly selected approximately 70 from all possible drug pairs and we performed 10,000

randomizations. For these 10,000 randomizations, 9,875 randomizations have more than one drug pair in the Overlapping Exposure category (p-value = 0.9875), while no randomization has more than 59 drug pairs in the Overlapping Exposure category (p-value = 0/10000 [$<10^{-4}$]). We performed the same permutation test for **Fig. 2** and **Supplementary Figs. 10-12**. For **Fig. 2**, to keep the same number of adverse drug interactions with known hypertensive drug combinations, we randomly selected approximately 70 adverse drug-drug pairs from 1,512 adverse drug-drug pairs related to high-blood pressure, using a bootstrapping algorithm in R software (https://cran.r-project.org/). We repeated this randomization process 100 times and provided the error bars (standard deviation) in the “adverse drug interactions” column of **Fig. 2**.

Supplementary Figures

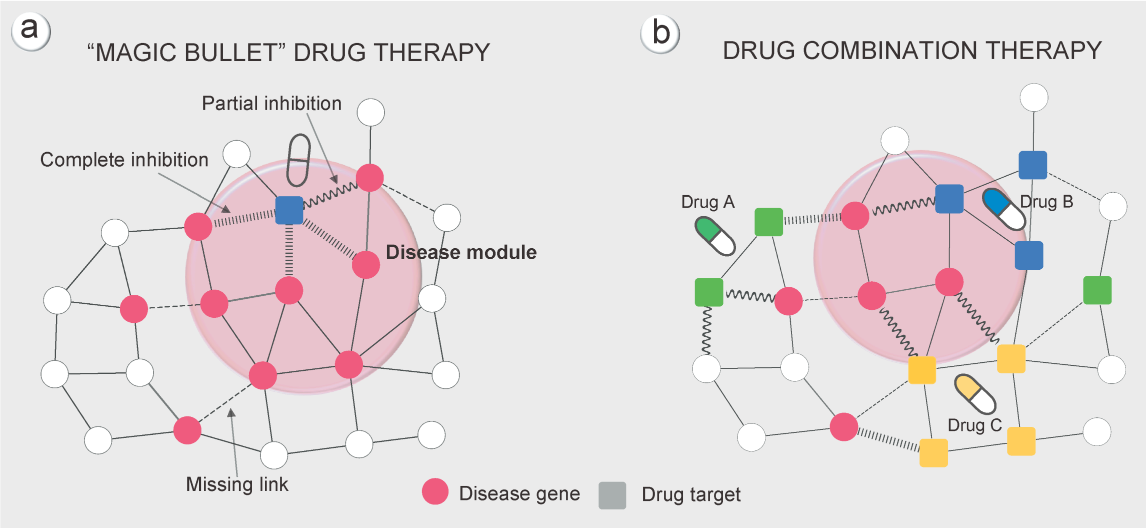

Supplementary Fig. 1 Proof-of-concept of identifying new drug combinations in the human protein-protein interactome. Based on the network pharmacology hypothesis[44], drug target proteins (representing nodes within cellular networks) are often intrinsically coupled to both therapeutic and adverse effects. (**a**) Traditional "magic bullet" drugs that design maximally selective ligands to target a single disease protein have attributed to a significant decrease in pharmaceutical research and development productivity due to lack of efficacy or toxicity effects[44]. (**b**) Drug combinations that show partial inhibition of a small set of disease proteins (i.e., the disease module) have been recognized to be more efficient than "magic bullet" drugs in monotherapies[45]. In this study, we hypothesize that quantifying the relationship between drug targets and disease proteins in the human interactome may lead to rational, network-based drug combination design strategies.

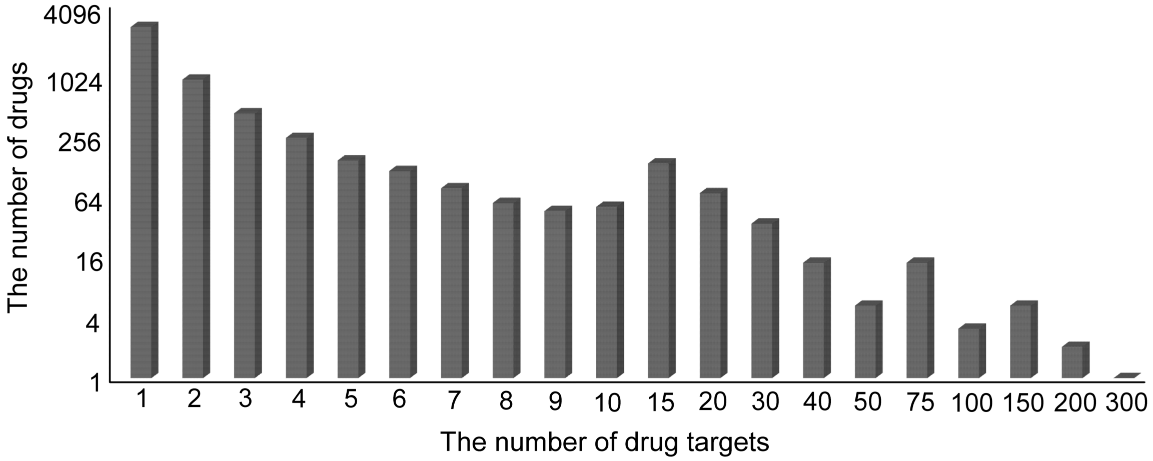

Supplementary Fig. 2 Distribution of drug degree (connectivity) in the drug-target interaction bipartite network. Histogram showing the number of targets (The average number of targets (degree) is approximate 3) for 4,428 FDA-approved or clinically investigational drugs that have at least one targets with the known experimental evidence (**Supplementary Data 2**). Histogram was illustrated based on Log2 scale.

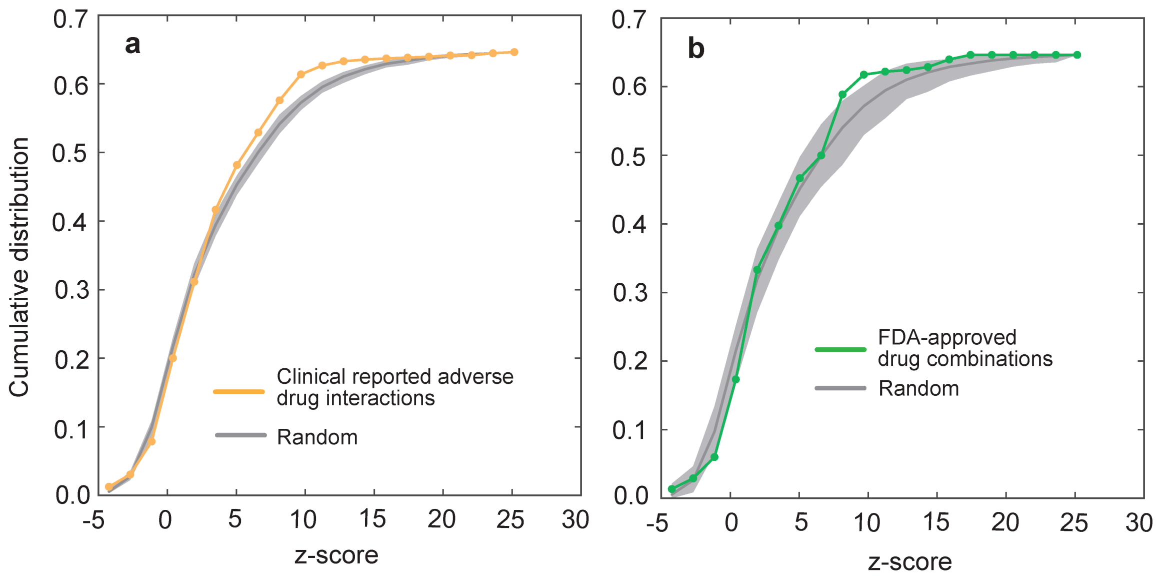

Supplementary Fig. 3 Network-based discrimination of drug-drug relationships by using the z-score (Supplementary Note 1) to quantify the network distance of drug targets in the human interactome. Curves showing the discrimination of clinically reported adverse drug-drug interactions (yellow, **a**), or FDA-approved or experimentally validated beneficial combinations (green, **b**) comparing to the randomly selected (not yet reported) drug pairs. Gray lines show the distribution of the same portion of randomly selected drug pairs as adverse drug-drug interactions or drug combination pairs. The gray range indicates the standard deviation over 200 random simulations.

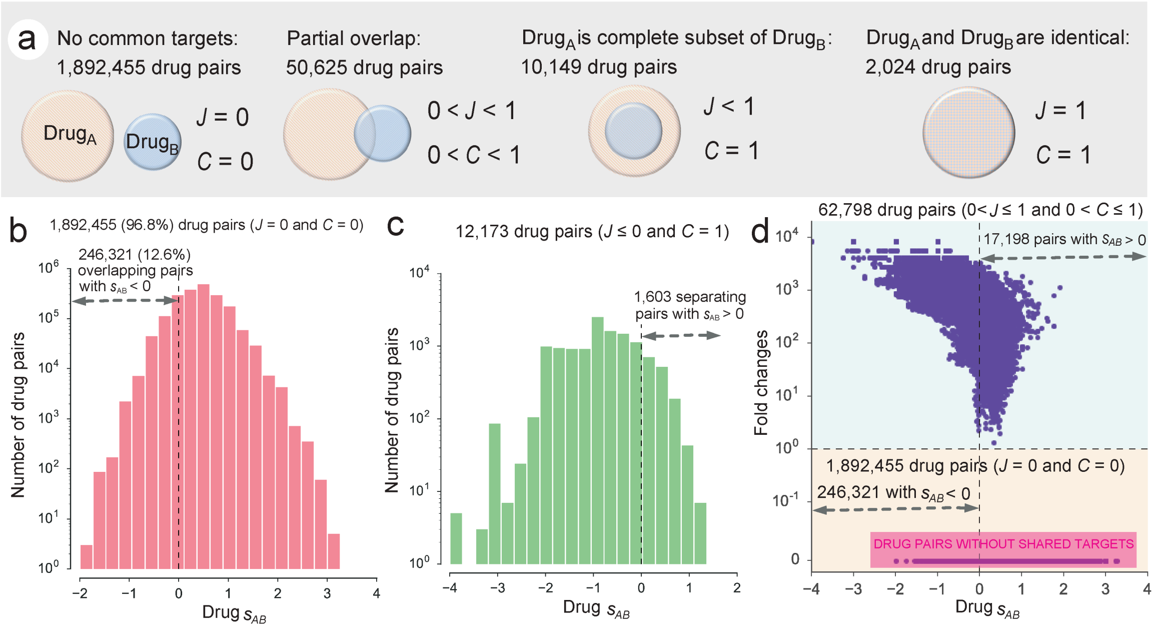

Supplementary Fig. 4 Comparison of network separation vs. target set-overlap approach. (**a** to **d**) The interplay between target-set overlapping and the network-based relationships between drug pairs. (**a**) The relationship between target sets of drugs A and B is captured by the overlapping coefficient $C = |A \cap B|/\min(|A|,|B|)$ and the Jaccard-index $J = |A \cap B|/|A \cup B|$. Approximately 96.8% (1,892,455/1,955,253) of drug pairs do not share targets (*J*=*C*=0); hence, their relationships cannot be identified via the shared targets (target-set overlapping approaches). (**b**) Distribution of network separation (*s*-score*)* for drug pairs without shared targets. We find that 246,321 (12.6%) drug pairs have overlapping modules ($s_{AB} < 0$), despite having disjoint target sets. (**c**) The distribution of s_{AB} for drug pairs with complete target overlapping (*C*=1) reveals a diversity of network-based relationships, including 1,603 separated drug modules ($s_{AB} > 0$). (**d**) Fold change of the number of shared targets compared to random expectation vs. for all drug pairs. 1,892,455 (96.8%) drug pairs without shared targets are highlighted with red background. For that shared at least one target, the target-overlap is larger than expected by chance. Interestingly, 17,198 drug pairs with shared targets are separated ($s_{AB} > 0$) in the human interactome network. Furthermore, a considerable number of 246,321 (12.6%) drug pairs without shared targets reveal detectable network overlap ($s_{AB} < 0$). Fold-change <1 indicates depletion (i.e., fewer common targets than expected), whereas fold-change >1 indicates enrichment as described in our previous study (Menche et al., Science 2015).

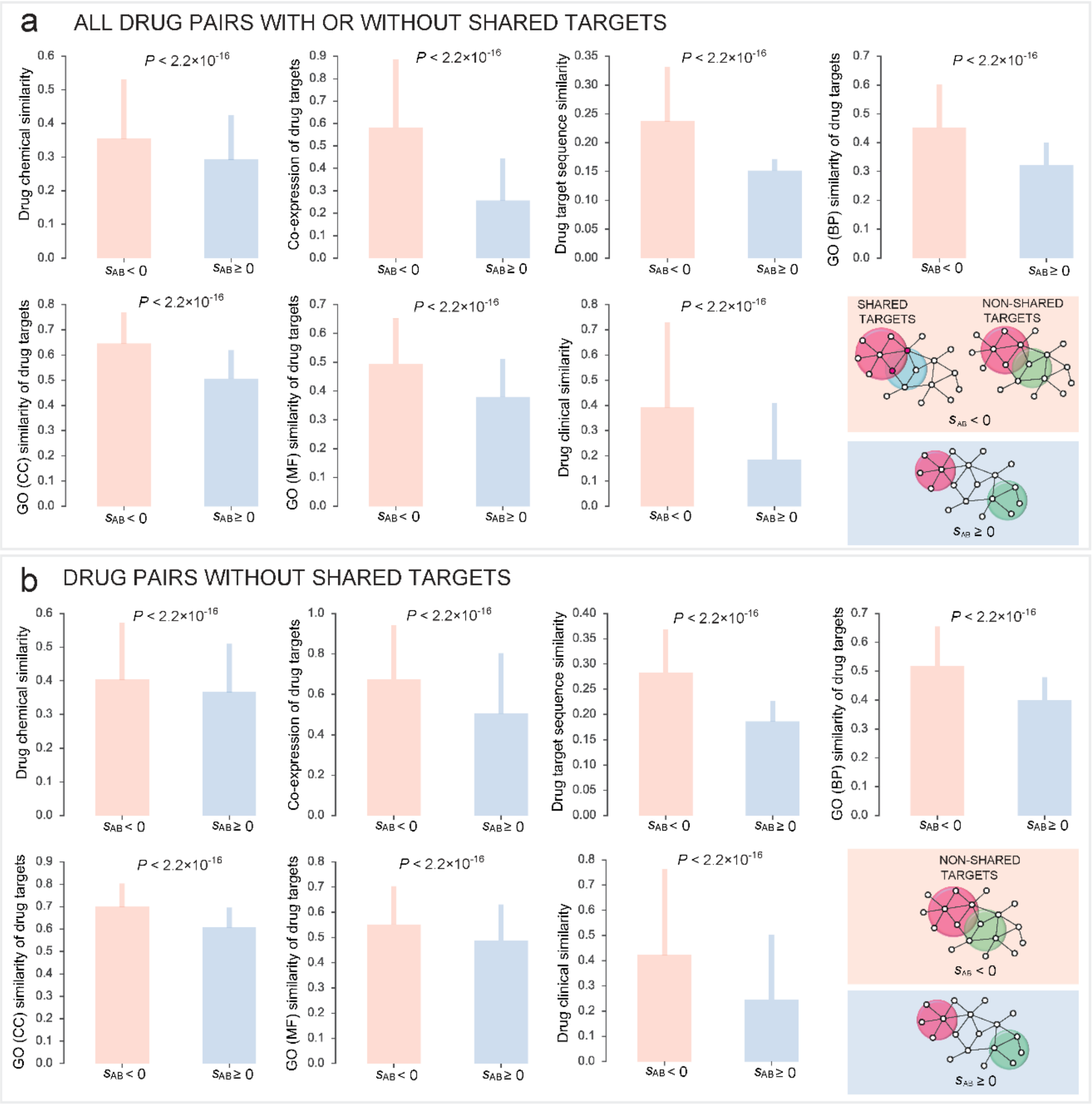

Supplementary Fig. 5 Network-based separation is independent from common targets shared by drug pairs. (**a**) Chemical, biological, functional, and clinical similarities for the overlapping drug pairs (s_{AB} < 0) vs. the separated drug pairs (s_{AB} ≥ 0) among all drug pairs with or without shared targets. (**b**) The same as (a) for drug pairs that do not share common targets (J=0 and C=0 in **Supplementary Fig. 4a**). Error bars indicate the standard deviation. The P is calculated by one-sided Wilcoxon signed-rank test. As shown in **Fig. 1d**, two drugs with very negative separation score have very low chemical similarity. A similar observation was made for cellular component similarity (**Fig. 1h**). Here, we find that drug pairs with strong separation score (low value) have higher chemical similarity and cellular component similarity. The apparent difference in those two measures (**Fig. 1d** and **1h**) out of 7 is merely due to fluctuations caused by undersampling. Indeed, less than 0.1% of the data points fall into the bins of very negative separation score (s-score [s_{AB}] < -2.0).

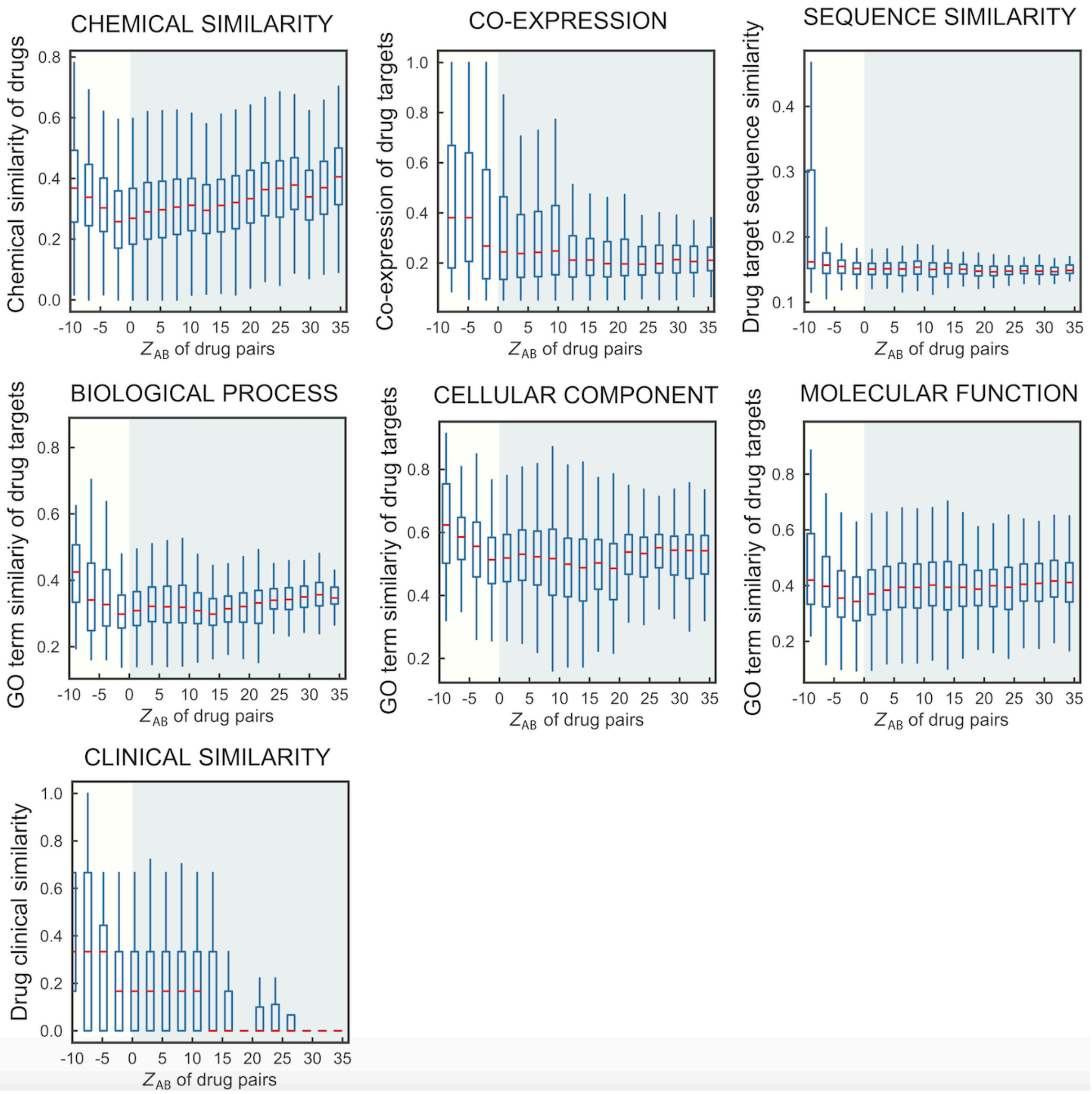

Supplementary Fig. 6 The interplay between z-scores of drug pairs and five types of drug profiles: drug-drug chemical similarity (CHEMICAL SIMILARITY); drug target-encoding gene co-expression pattern across human tissues (CO-EXPRESSION); drug target protein sequence similarity (SEQUENCE SIMILARITY); the Gene Ontology (GO) annotations, we determine for each drug how similar its associated target-encoding genes are in terms of their biological processes (BIOLOGICAL PROCESS), cellular component (CELLULAR COMPONENT), and molecular function (MOLECULAR FUNCTION); and clinical (therapeutic) similarity (CLINICAL SIMILARITY) derived from Anatomical Therapeutic Chemical Classification Systems codes (see Methods). Overlapping drug pairs are highlighted in white background ($s_{AB} < 0$); topologically separated drug pairs are highlighted in blue background ($s_{AB} \geq 0$).

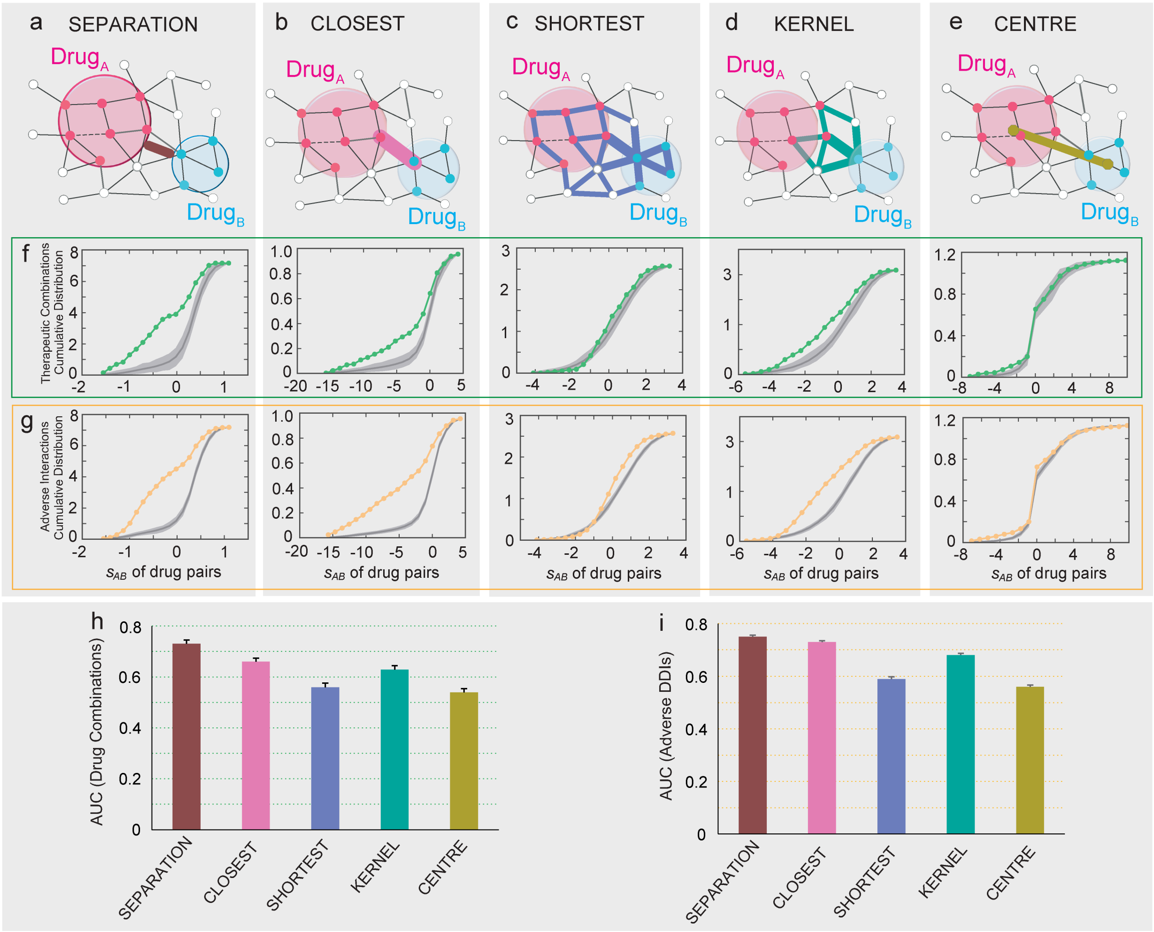

Supplementary Fig. 7 Validating network proximity-based stratification of drug-drug combinations. (**a** to **f**) Cartoon diagrams of five different network-based topological distance measures: (**a**) separation, (**b**) closest, (**c**) shortest, (**d**) kernel, and (**e**) centre (see Methods). Both FDA-approved or experimentally validated pairwise drug combinations (green lines, **f**) and clinically reported adverse drug interactions (yellow lines, **g**) have a higher network proximity (low s_{AB}) compared to the same number of random pairs. Gray lines show the distribution of the same portion of randomly selected drug pairs as the matching number of adverse drug interactions or drug combination pairs. Gray shadow denotes the standard derivation over 100 random simulations. The area under the receiver operating characteristic curve (AUC) for discrimination of FDA-approved or experimentally validated therapeutic combinations (**h**) and clinically reported adverse drug interactions (**i**) from the randomly selected drug pairs by five different network-based measures.

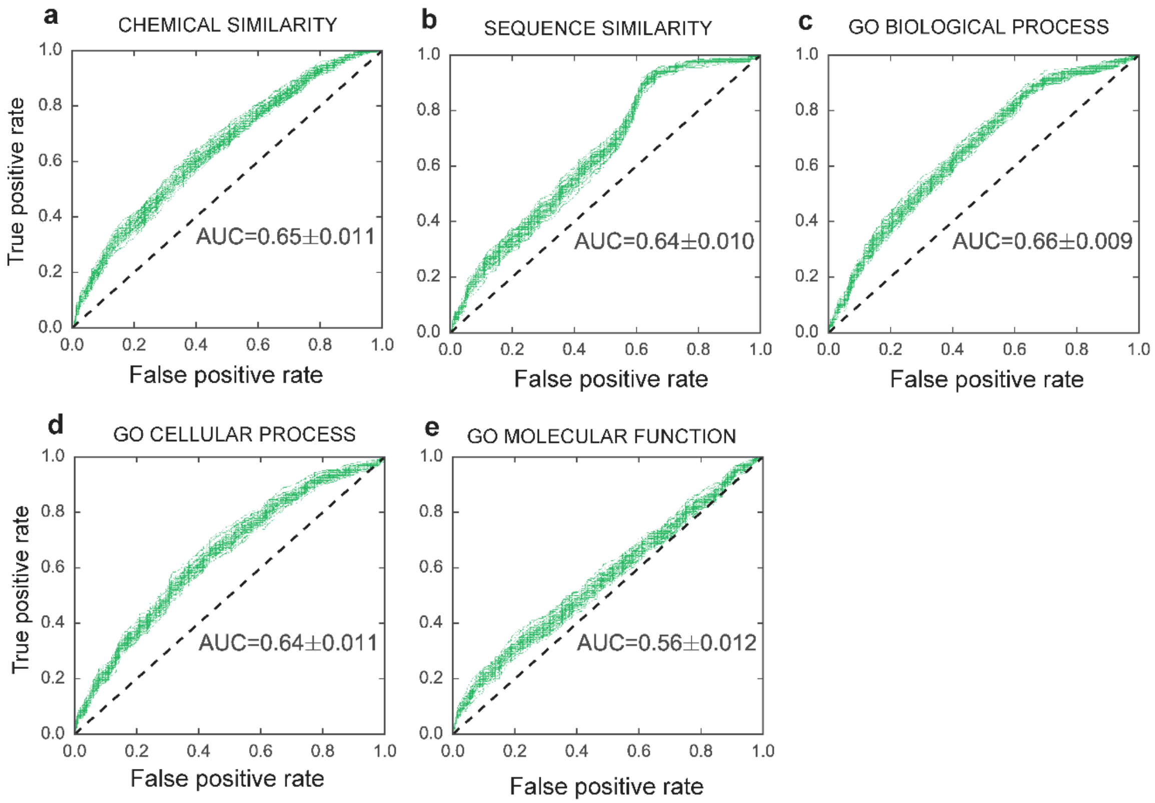

Supplementary Fig. 8 Receiver Operating Characteristic curves for discrimination of FDA-approved or experimentally validated therapeutic drug combinations (**Supplementary Note 3** and **Supplementary Data 1**) from the randomly selected drug pairs using chemoinformatics and bioinformatics approaches. AUC: The area under receiver operating characteristic curve. The description of chemoinformatics and bioinformatics approaches (**a-e**) is provided in Methods.

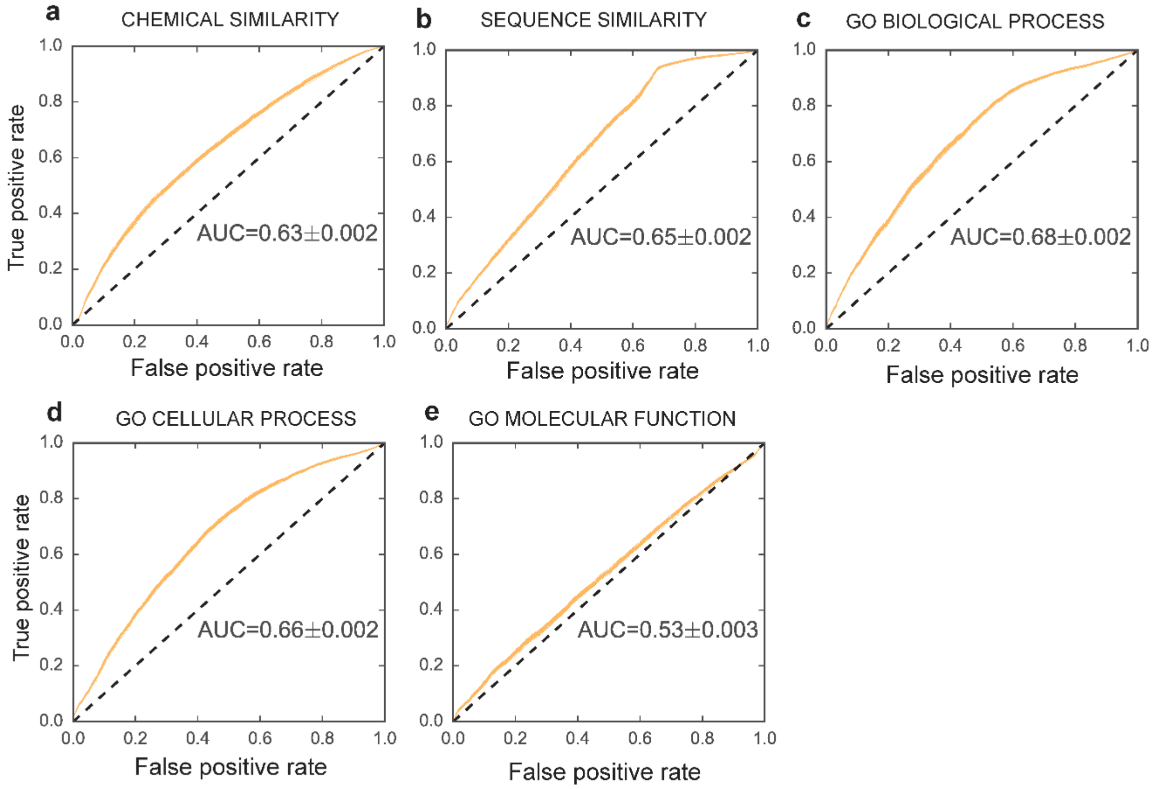

Supplementary Fig. 9 Receiver operating characteristic curves for discrimination of clinically reported adverse drug interactions (**Supplementary Data 4**) from the randomly selected drug pairs using chemoinformatics and bioinformatics approaches. AUC: The area under receiver operating characteristic curve. The description of chemoinformatics and bioinformatics approaches (**a-e**) is provided in Methods.

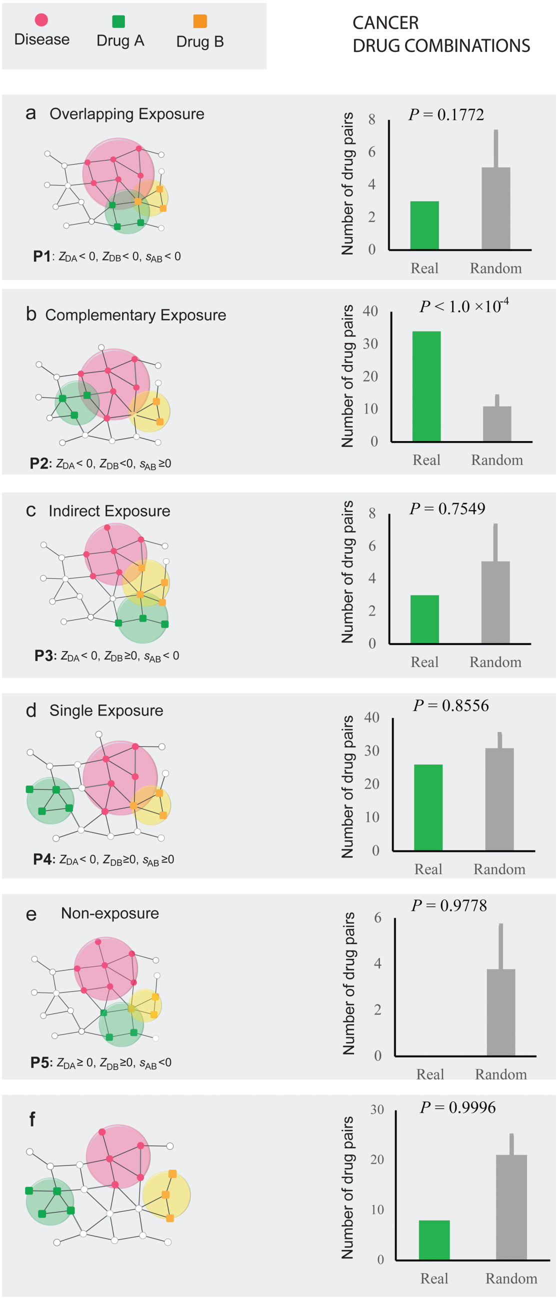

Supplementary Fig. 10 Network architecture of approved, clinically, or pre-clinically validated anticancer drug combinations (**Supplementary Data 3**). Schematic diagrams of the six distinct classes (**a-f**) capturing the network-based relationship between targets of two drugs and disease module proteins (drug-drug-disease). Color histograms (Real) show the validated cancer drug combinations across *P1-P6*. Gray boxes (Random) show random expectation. Error bars indicate the standard deviation. The p-value (*P*) is calculated by testing 10,000 permutations. The detailed definition of six possible network architectures (**a-f**) are provided in **Fig. 2.** Cancer is a chronic disease with a strong genetic contribution, rarely caused by adverse drug-drug interactions. We illustrated the anticancer drug combinations only.

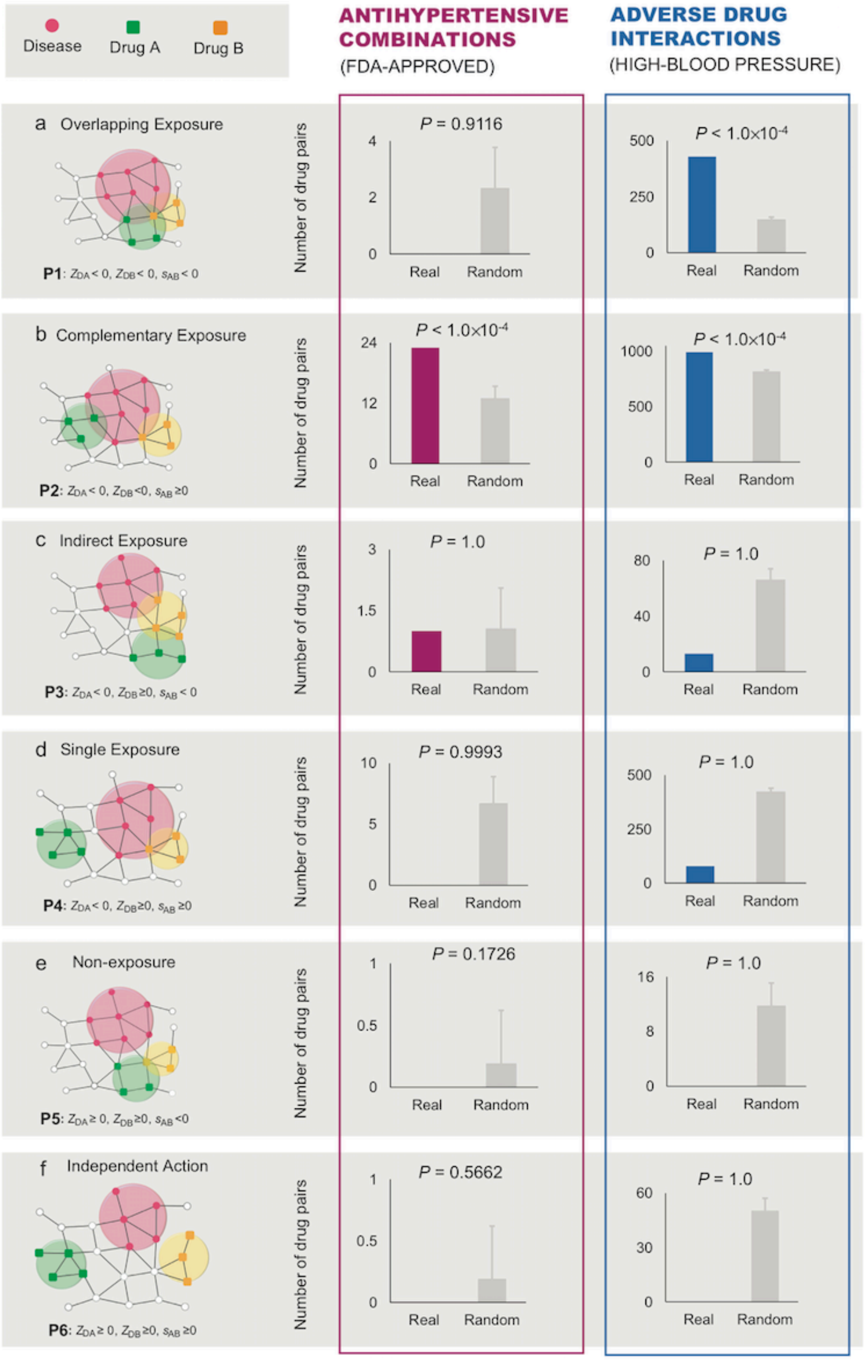

Supplementary Fig. 11 The efficacy of FDA-approved hypertensive drug-drug interactions only. **a-f**, Schematic diagrams of the six distinct classes capturing the network-based relationship between two drug-target modules and one disease module on a drug-drug-disease combination. Color histograms (Real) show the FDA-approved antihypertensive combinations (purple) and clinically reported adverse drug interactions on high-blood pressure (blue), respectively. Gray boxes (Random) show random expectation. Error bars indicate the standard deviation. The p-value (*P*) is calculated by testing 10,000 permutations.

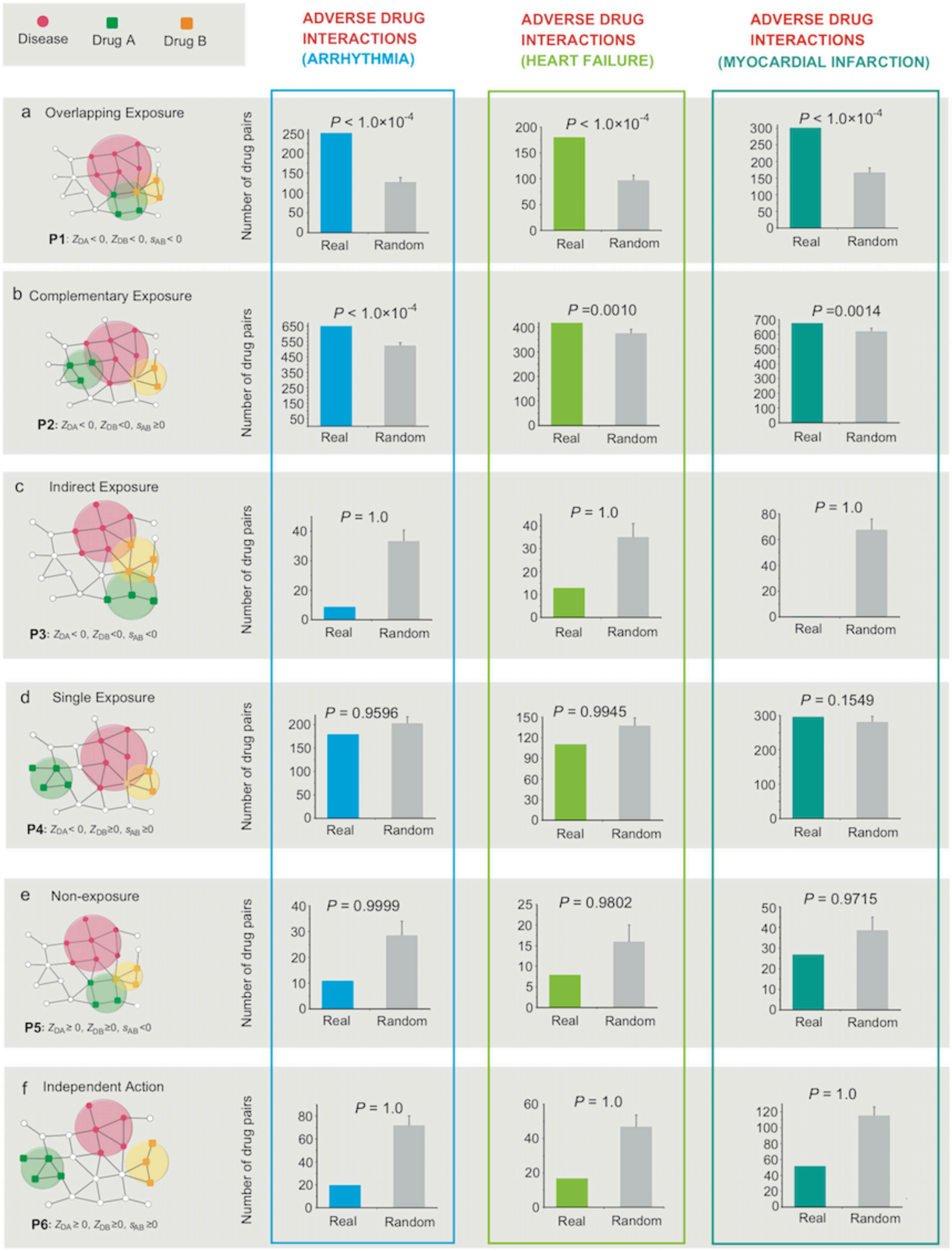

Supplementary Fig. 12 Network architecture of clinically reported adverse drug-drug interaction pairs in three cardiovascular outcomes: arrhythmia, heart failure, and myocardial infarction. Schematic diagrams of the six distinct classes (**a-f**) capturing the network-based relationship between targets of two drugs and disease module proteins (drug-drug-disease). Color histograms (Real) show the clinically reported adverse drug-drug interaction pairs across *P1-P6*. Gray boxes (Random) show random expectation. Error bars indicate the standard deviation. The p-value (*P*) is calculated by testing 10,000 permutations.

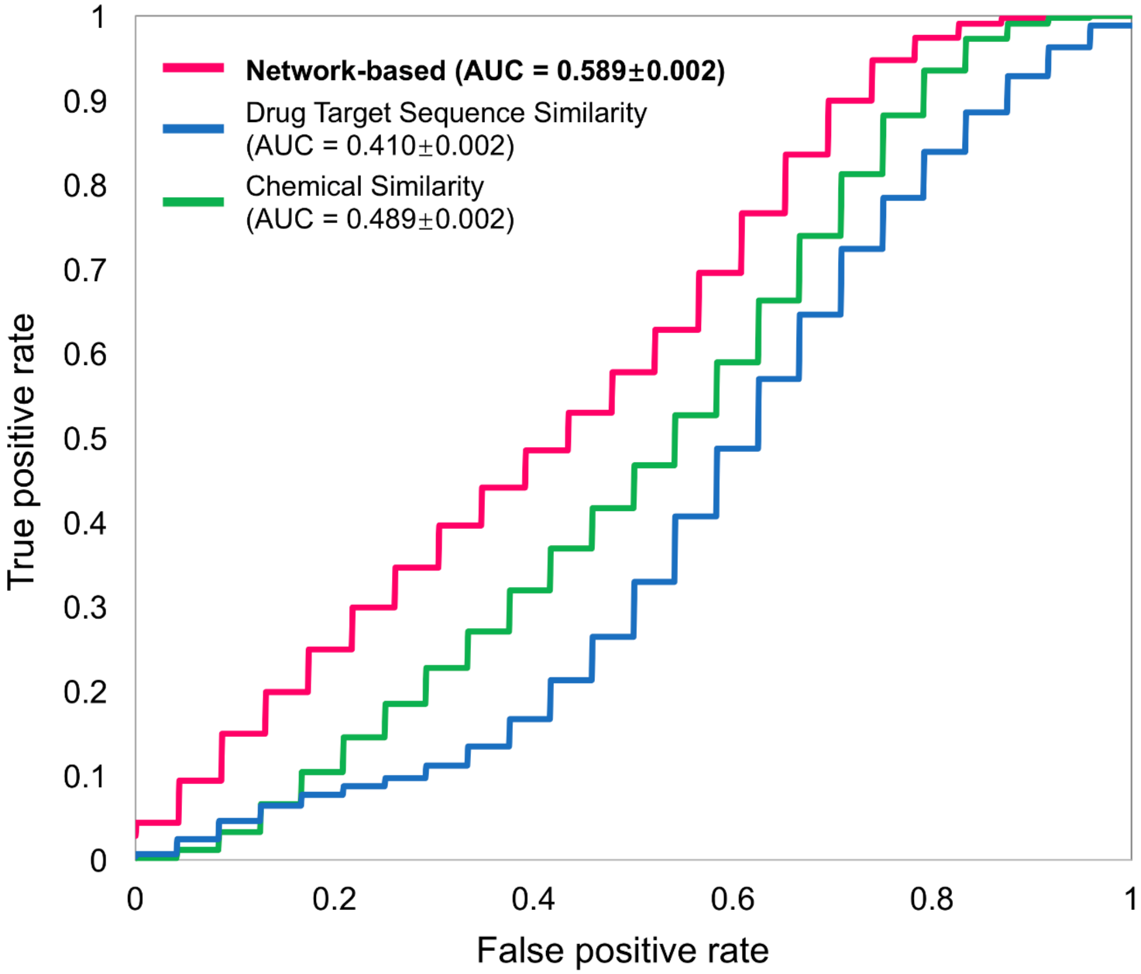

Supplementary Fig. 13 Receiver operating characteristic curves for predicting new hypertensive drug combinations using our network-based model, chemoinformatics, and bioinformatics approaches, respectively. In total, 24 FDA approved drug combinations for treatment of hypertension (**Supplementary Data 3**) were used as an external validation set. AUC: The area under receiver operating characteristic curve.

Supplementary Table 1. Top 30 network-predicted combinations for hydrochlorothiazide in treatment of hypertension.

#	Drug A	Drug B	Network Proximity	Network Principle	References
1	Hydrochlorothiazide	Etacrynic acid	0.013	P2	NA
2	Hydrochlorothiazide	Irbesartan	0.054	P2	a&b
3	Hydrochlorothiazide	Guanfacine	0.067	P2	NA
4	Hydrochlorothiazide	Nitrendipine	0.101	P2	[46]
5	Hydrochlorothiazide	Isradipine	0.110	P2	NA
6	Hydrochlorothiazide	Amlodipine	0.131	P2	a&b
7	Hydrochlorothiazide	Nifedipine	0.140	P2	[47]
8	Hydrochlorothiazide	Mibefradil	0.277	P2	[48]
9	Hydrochlorothiazide	Felodipine	0.280	P2	[49]
10	Hydrochlorothiazide	Nebivolol	0.285	P2	[50]
11	Hydrochlorothiazide	Nisoldipine	0.300	P2	NA
12	Hydrochlorothiazide	Acebutolol	0.317	P2	a
13	Hydrochlorothiazide	Atenolol	0.317	P2	a&b
14	Hydrochlorothiazide	Captopril	0.329	P2	a
15	Hydrochlorothiazide	Bevantolol	0.362	P2	NA
16	Hydrochlorothiazide	Telmisartan	0.362	P2	b
17	Hydrochlorothiazide	Bosentan	0.400	P2	NA
18	Hydrochlorothiazide	Verapamil	0.415	P2	[51]
19	Hydrochlorothiazide	Amiloride	0.421	P2	[52]
20	Hydrochlorothiazide	Prazosin	0.489	P2	[53]
21	Hydrochlorothiazide	Phenoxybenzamine	0.535	P2	NA
22	Hydrochlorothiazide	Bethanidine	0.543	P2	NA
23	Hydrochlorothiazide	Nicardipine	0.547	P2	[54]
24	Hydrochlorothiazide	Phentolamine	0.550	P2	NA
25	Hydrochlorothiazide	Methyldopa	0.567	P2	[52]
26	Hydrochlorothiazide	Moexipril	0.567	P2	[52]
27	Hydrochlorothiazide	Spironolactone	0.588	P2	[52]
28	Hydrochlorothiazide	Bisoprolol	0.695	P2	[52]
29	Hydrochlorothiazide	Metoprolol	0.695	P2	[52]
30	Hydrochlorothiazide	Timolol	0.695	P2	[52]

Note: In total, 21 predicted ones (70% success rate) are validated by FDA-approved evidence, clinical studies from Clinicaltrials.gov, or previously reported preclinical data [a]FDA-approved; [b]Clinicaltrials.gov. NA: There are non-available literature, preclinical, or clinical data for the prediction.

Supplementary References

1. Guney, E., Menche, J., Vidal, M. & Barabasi, A. L. Network-based in silico drug efficacy screening. *Nat. Commun.* **7**, 10331 (2016).
2. Rolland, T. et al. A proteome-scale map of the human interactome network. *Cell* **159**, 1212-1226 (2014).
3. Rual, J. F. et al. Towards a proteome-scale map of the human protein-protein interaction network. *Nature* **437**, 1173-1178 (2005).
4. Cheng, F., Jia, P., Wang, Q. & Zhao, Z. Quantitative network mapping of the human kinome interactome reveals new clues for rational kinase inhibitor discovery and individualized cancer therapy. *Oncotarget* **5**, 3697-3710 (2014).
5. Peri, S. et al. Human protein reference database as a discovery resource for proteomics. *Nucleic Acids Res.* **32**, D497-501 (2004).
6. Newman, R. H. et al. Construction of human activity-based phosphorylation networks. *Mol. Syst. Biol.* **9**, 655 (2013).
7. Hu, J. et al. PhosphoNetworks: a database for human phosphorylation networks. *Bioinformatics* **30**, 141-142 (2014).
8. Hornbeck, P. V. et al. PhosphoSitePlus, 2014: mutations, PTMs and recalibrations. *Nucleic Acids Res.* **43**, D512-520 (2015).
9. Lu, C. T. et al. DbPTM 3.0: an informative resource for investigating substrate site specificity and functional association of protein post-translational modifications. *Nucleic Acids Res.* **41**, D295-305 (2013).
10. Dinkel, H. et al. Phospho.ELM: a database of phosphorylation sites--update 2011. *Nucleic Acids Res.* **39**, D261-267 (2011).
11. Chatr-Aryamontri, A. et al. The BioGRID interaction database: 2015 update. *Nucleic Acids Res.* **43**, D470-478 (2015).

12. Cowley, M. J. et al. PINA v2.0: mining interactome modules. *Nucleic Acids Res.* **40**, D862-865 (2012).
13. Licata, L. et al. MINT, the molecular interaction database: 2012 update. *Nucleic Acids Res.* **40**, D857-861 (2012).
14. Orchard, S. et al. The MIntAct project--IntAct as a common curation platform for 11 molecular interaction databases. *Nucleic Acids Res.* **42**, D358-363 (2014).
15. Breuer, K. et al. InnateDB: systems biology of innate immunity and beyond--recent updates and continuing curation. *Nucleic Acids Res.* **41**, D1228-1233 (2013).
16. Meyer, M. J., Das, J., Wang, X. & Yu, H. INstruct: a database of high-quality 3D structurally resolved protein interactome networks. *Bioinformatics* **29**, 1577-1579 (2013).
17. Fazekas, D. et al. SignaLink 2 - a signaling pathway resource with multi-layered regulatory networks. *BMC Syst. Biol.* **7**, 7 (2013).
18. Coordinators, N. R. Database resources of the National Center for Biotechnology Information. *Nucleic Acids Res.* **44**, D7-19 (2016).
19. Menche, J. et al. Disease networks. Uncovering disease-disease relationships through the incomplete interactome. *Science* **347**, 1257601 (2015).
20. Liu, Y. et al. DCDB 2.0: a major update of the drug combination database. *Database* **2014**, bau124 (2014).
21. Yang, H. et al. Therapeutic target database update 2016: enriched resource for bench to clinical drug target and targeted pathway information. *Nucleic Acids Res.* **44**, D1069-1074 (2016).
22. Kalra, S., Kalra, B. & Agrawal, N. Combination therapy in hypertension: An update. *Diabetol. Metab. Syndr.* **2**, 44 (2010).

23. Sica, D. A. Drug combinations in the treatment of hypertension: never-ending novelty. *Hypertension* **56**, 22-23 (2010).
24. Huang, H., Zhang, P., Qu, X. A., Sanseau, P. & Yang, L. Systematic prediction of drug combinations based on clinical side-effects. *Sci. Rep.* **4**, 7160 (2014).
25. Charpentier, G. Oral combination therapy for type 2 diabetes. *Diabetes Metab Res. Rev.* **18 Suppl 3**, S70-76 (2002).
26. Bennett, W. L. et al. Comparative effectiveness and safety of medications for type 2 diabetes: an update including new drugs and 2-drug combinations. *Ann. Intern. Med.* **154**, 602-613 (2011).
27. Group, A. S. et al. Effects of combination lipid therapy in type 2 diabetes mellitus. *N. Engl. J. Med.* **362**, 1563-1574 (2010).
28. Crystal, A. S. et al. Patient-derived models of acquired resistance can identify effective drug combinations for cancer. *Science* **346**, 1480-1486 (2014).
29. Holbeck, S. L. et al. The National Cancer Institute ALMANAC: A comprehensive screening resource for the detection of anticancer drug pairs with enhanced therapeutic activity. *Cancer Res.* **77**, 3564-3576 (2017).
30. Held, M. A. et al. Genotype-selective combination therapies for melanoma identified by high-throughput drug screening. *Cancer Discovery* **3**, 52-67 (2013).
31. Roller, D. G. et al. Synthetic lethal screening with small-molecule inhibitors provides a pathway to rational combination therapies for melanoma. *Mol. Cancer Therapeut.* **11**, 2505-2515 (2012).

32. Friedman, A. A. et al. Landscape of targeted anti-cancer drug synergies in melanoma identifies a novel BRAF-VEGFR/PDGFR combination treatment. *PLoS One* **10**, e0140310 (2015).
33. Bodenreider, O. The Unified Medical Language System (UMLS): integrating biomedical terminology. *Nucleic Acids Res.* **32**, D267-270 (2004).
34. Law, V. et al. DrugBank 4.0: shedding new light on drug metabolism. *Nucleic Acids Res.* **42**, D1091-1097 (2014).
35. Amberger, J. S., Bocchini, C. A., Schiettecatte, F., Scott, A. F. & Hamosh, A. OMIM.org: Online Mendelian Inheritance in Man (OMIM(R)), an online catalog of human genes and genetic disorders. *Nucleic Acids Res.* **43**, D789-798 (2015).
36. Davis, A. P. et al. The Comparative Toxicogenomics Database's 10th year anniversary: update 2015. *Nucleic acids research* **43**, D914-920 (2015).
37. Landrum, M. J. et al. ClinVar: public archive of relationships among sequence variation and human phenotype. *Nucleic Acids Res.* **42**, D980-985 (2014).
38. Welter, D. et al. The NHGRI GWAS Catalog, a curated resource of SNP-trait associations. *Nucleic Acids Res.* **42**, D1001-1006 (2014).
39. Li, M. J. et al. GWASdb v2: an update database for human genetic variants identified by genome-wide association studies. *Nucleic Acids Res.* **44**, D869-876 (2016).
40. Denny, J. C. et al. Systematic comparison of phenome-wide association study of electronic medical record data and genome-wide association study data. *Nat. Biotechnol.* **31**, 1102-1110 (2013).
41. Yu, W., Gwinn, M., Clyne, M., Yesupriya, A. & Khoury, M. J. A navigator for human genome epidemiology. *Nat. Genet.* **40**, 124-125 (2008).

42. Pinero, J. et al. DisGeNET: a discovery platform for the dynamical exploration of human diseases and their genes. *Database* **2015**, bav028 (2015).
43. Hothorn, T., K. Hornik, M.A. van de Wiel, and A. Zeileis. 2015. Implementing a Class of Permutation Tests: The coin Package. cran.r-project.org/web/packages/coin/vignettes/coin_implementation.pdf.
44. Hopkins, A. L. Network pharmacology: the next paradigm in drug discovery. *Nat. Chem. Biol.* **4**, 682-690 (2008).
45. Csermely, P., Agoston, V. & Pongor, S. The efficiency of multi-target drugs: the network approach might help drug design. *Trends Pharmacol. Sci.* **26**, 178-182 (2005).
46. Han, P., Chu, Z. X., Shen, F. M., Xie, H. H. & Su, D. F. Synergism of hydrochlorothiazide and nitrendipine on reduction of blood pressure and blood pressure variability in spontaneously hypertensive rats. *Acta Pharmacol. Sin.* **27**, 1575-1579 (2006).
47. Xie, H. H., Zhang, X. F., Chen, Y. Y., Shen, F. M. & Su, D. F. Synergism of hydrochlorothiazide and nifedipine on blood pressure variability reduction and organ protection in spontaneously hypertensive rats. *Hypertension Res.* **31**, 685-691 (2008).
48. Carney, S. et al. The addition of mibefradil to chronic hydrochlorothiazide therapy in hypertensive patients is associated with a significant antihypertensive effect. *J. Hum. Hypertens.* **11**, 459-466 (1997).
49. Koenig, W. et al. Effects of felodipine ER and hydrochlorothiazide on blood rheology in essential hypertension--a randomized, double-blind, crossover study. *J. Intern. Med.* **229**, 533-538 (1991).
50. Malacco, E. Nebivolol/hydrochlorothiazide (HCTZ) combination in patients with essential hypertension: a pooled analysis from five non-interventional

studies with a focus on diabetic and elderly patients. *Euro. Rev. Med. Pharmacol. Sci.* **14**, 427-434 (2010).

51. Holzgreve, H., Distler, A., Michaelis, J., Philipp, T. & Wellek, S. Verapamil versus hydrochlorothiazide in the treatment of hypertension: results of long term double blind comparative trial. Verapamil versus Diuretic (VERDI) Trial Research Group. *BMJ* **299**, 881-886 (1989).
52. Skolnik, N. S., Beck, J. D. & Clark, M. Combination antihypertensive drugs: recommendations for use. *Am. Fam. Physician* **61**, 3049-3056 (2000).
53. Pitkajarvi, T., Kyostila, S., Kontro, J. & Mattila, M. J. Antihypertensive drug combinations: prazosin, hydrochlorothiazide and clonidine. *Ann. Clin. Res.* **9**, 296-300 (1977).
54. Fagan, T. C. et al. Nicardipine and hydrochlorothiazide in essential hypertension. *Clin. Pharm. Therapeut.* **45**, 429-438 (1989).
