# Supplementary material for: Network-based prediction of drug combinations
Source: Nat Commun. 2019 Mar 13;10:1197. doi: 10.1038/s41467-019-09186-x (PMC6416394; doi:10.1038/s41467-019-09186-x)
Supplement: Supplementary file 2 — Description of Additional Supplementary Files [file 41467_2019_9186_MOESM2_ESM.docx]

**Description of Additional Supplementary Files**

File Name: Supplementary Data 1

Description: The human protein-protein interactome used in our study.

File Name: Supplementary Data 2

Description: The experimentally validated drug-target interactions.

File Name: Supplementary Data 3

Description: The lists of experimentally validated drug combinations.

File Name: Supplementary Data 4

Description: The lists of clinically reported adverse drug interactions.

File Name: Supplementary Data 5

Description: The network-predicted hypertensive drug combinations.
